# Real-world data on the incidence and risk of Guillain–Barré syndrome following SARS-CoV-2 vaccination: a prospective surveillance study

**DOI:** 10.1038/s41598-023-30940-1

**Published:** 2023-03-07

**Authors:** Jongmok Ha, Suyeon Park, Hyunwook Kang, Taeeun Kyung, Namoh Kim, Dong Kyu Kim, Hyeonjoon Kim, Kihoon Bae, Min Cheol Song, Kwang June Lee, Euiho Lee, Beom Seuk Hwang, Jinyoung Youn, Jin Myoung Seok, Kunhee Park

**Affiliations:** 1 Infectious Disease Control Center, Gyeonggi Provincial Government, Suwon, Korea; 2 Department of Biostatistics, Soonchunhyang University Seoul Hospital, Seoul, Korea; 3 Department of Neurology, Samsung Medical Center, Sungkyunkwan University School of Medicine, 81 Irwon-Ro Gangnam-Gu, Seoul, 06351 Korea; 4 Neuroscience Center, Samsung Medical Center, Seoul, Korea; 5 Department of Neurology, Soonchunhyang University Hospital Cheonan, Soonchunhyang University College of Medicine, Cheonan, Korea; 6 Department of Applied Statistics, Chung-Ang University, Seoul, Republic of Korea

**Keywords:** Diseases of the nervous system, Neurology, Neurological disorders, Neuromuscular disease

## Abstract

Increasing evidence suggests an association between SARS-CoV-2 vaccines and Guillain–Barré syndrome (GBS). Nevertheless, little is understood about the contributing risk factors and clinical characteristics of GBS post SARS-CoV-2 vaccination. In this prospective surveillance study of 38,828,691 SARS-CoV-2 vaccine doses administered from February 2021 to March 2022 in the Gyeonggi Province, South Korea, 55 cases of GBS were reported post vaccination. We estimated the incidence rate of GBS per million doses and the incidence rate ratio for the vaccine dose, mechanism, age, and sex. Additionally, we compared the clinical characteristics of GBS following mRNA-based and viral vector-based vaccinations. The overall incidence of GBS following SARS-CoV-2 vaccination was 1.42 per million doses. Viral vector-based vaccines were associated with a higher risk of GBS. Men were more likely to develop GBS than women. The third dose of vaccine was associated with a lower risk of developing GBS. Classic sensorimotor and pure motor subtypes were the predominant clinical subtypes, and demyelinating type was the predominant electrodiagnostic subtype. The initial dose of viral-vector based vaccine and later doses of mRNA-based vaccine were associated with GBS, respectively. GBS following SARS-CoV-2 vaccination may not be clinically distinct. However, physicians should pay close attention to the classic presentation of GBS in men receiving an initial dose of viral vector-based SARS-CoV-2 vaccines.

## Introduction

As the struggle to overcome the aftermath of the COVID-19 global pandemic continues, nearly 12 billion doses of SARS-CoV-2 vaccines have been administered worldwide as of June 2022^1^; currently, we are faced with the consequences of mass vaccination, both developed and deployed at an unprecedented speed.

**Figure 1:**
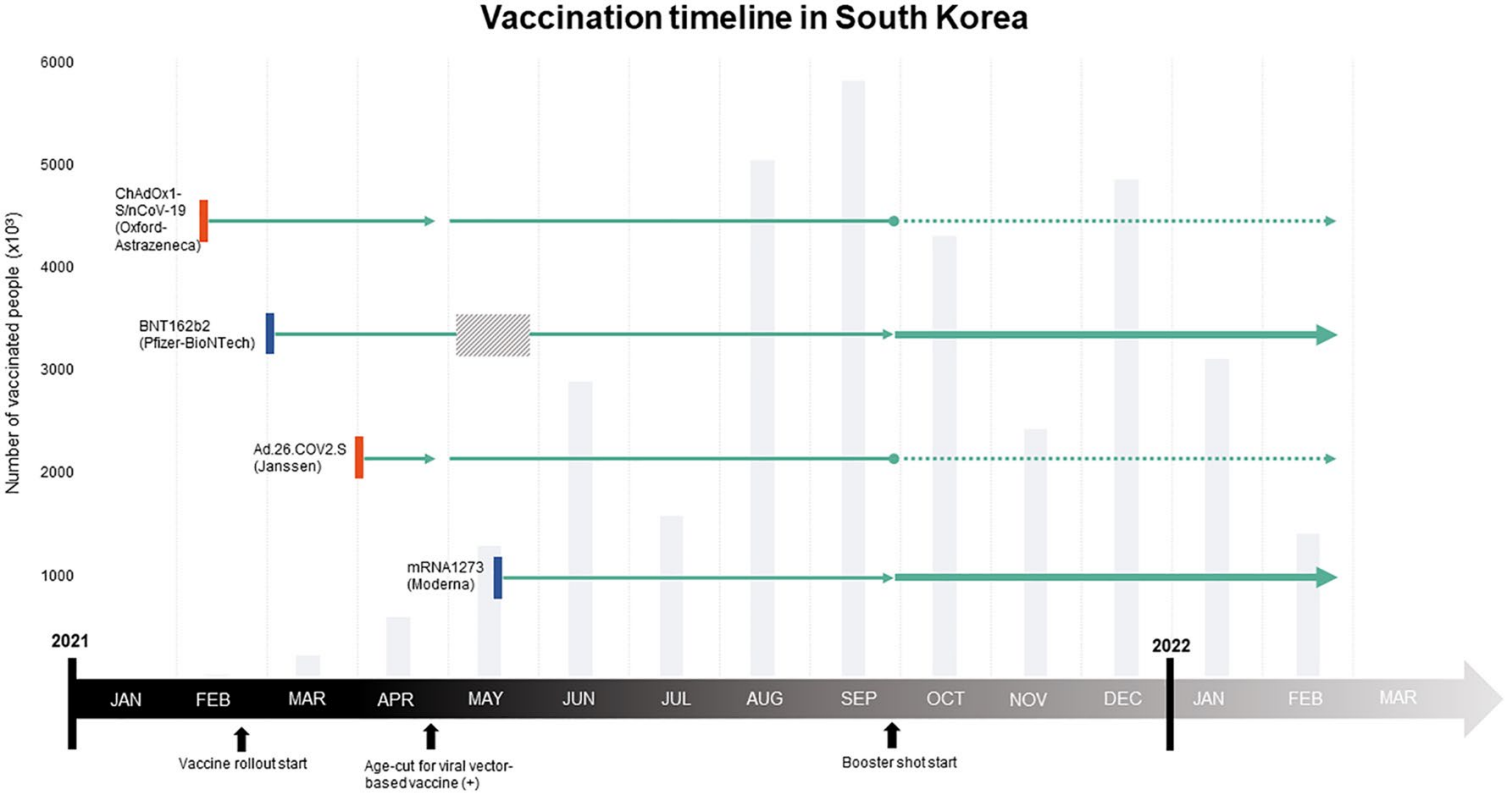
Vaccination timeline in South Korea; approval of four different types of vaccines; time-series of number of vaccinated people; and reported adverse events in the Gyeonggi province. A nationwide vaccination campaign for SARS-CoV-2 in South Korea was initiated on February 26th, 2021. Vaccines from four different manufacturers were approved: the viral vector-based ChAdOx1-S/nCoV-19 (Oxford-Astrazeneca) vaccine on February 10th, 2021; the BNT162b2 (Pfizer-BioNTech) vaccine on March 5th, 2021; the Ad26. COV2.S (Janssen) vaccine on April 7th, 2021; and the mRNA-1273 (Moderna) vaccine on May 21st, 2021. The initiation of each vaccine is represented by red and blue markers (red indicates mRNA-based and blue indicates viral vector-based). The slashed square area represents a temporary halt in the BNT162b2 vaccination in May 2021, owing to problems with vaccine supply. From the beginning of October, booster shots were given; most were mRNA vaccines (bold green line and arrow), and viral-vector vaccines (third dose of ChAdOx1-S/nCoV-19, an additional dose of Ad26.COV2.S) only represented a minority of vaccinated people (green dotted line and arrow). The gray bars in the background represent the total number of vaccinated people in the Gyeonggi province, South Korea, in the respective months.

The nationwide SARS-CoV-2 vaccine rollout in South Korea began in February 2021. Vaccines from four different manufacturers were approved sequentially: viral vector-based ChAdOx1-S/nCoV-19 (Oxford-Astrazeneca), Ad26. COV2.S (Janssen), mRNA-based BNT162b2 (Pfizer-BioNTech), and mRNA-1273 (Moderna) vaccines (Fig. 1). Due to thrombotic thrombocytopenia syndrome (TTS) following ChAdOx1-S/nCoV-19^2^ and Ad26. COV2.S^3^ vaccines, viral vector-based SARS-CoV-2 vaccines have been contraindicated in South Korea for individuals under 30 years of age since April 2021.

To address the growing safety concerns regarding SARS-CoV-2 vaccination, the provincial governments of South Korea have cooperated with the Korea Disease Control and Prevention Agency (KDCA) to gather data on adverse events following immunization (AEFI) and adverse events of special interest (AESI), including Guillain–Barré syndrome (GBS). Additionally, pharmacovigilance review meetings were held by interdisciplinary experts to discuss the link between vaccination and potential adverse events.

GBS is an acute-onset, progressive, monophasic, immune-mediated peripheral neuropathy that is frequently associated with diverse antecedent respiratory and gastrointestinal infections^4,5^. Historically, the potential link between GBS and vaccination (e.g. influenza, MMR, hepatitis B, diphtheria, etc.) has been addressed in multiple studies over decades^6–10^; influenza A (H1N1) in particular, has been postulated to be associated with an increased risk of GBS in some occasions^11,12^. However, there has been an increasing body of evidence suggesting an association between viral vector-based SARS-CoV-2 vaccines and GBS^13,14^. mRNA-based vaccines have also been suspected to have an association^15–19^, but controversies have remained^14,20^.

While studies thus far have focused profoundly on reviewing individual cases or analyzing large patient databases, only few studies have simultaneously gathered large population data and meticulously reviewed individually reported GBS cases from a clinical perspective. With limited data, policymakers and physicians struggle to recommend the most suitable vaccine for individual recipients, who may be at an increased risk for certain vaccines. Hence, to tip the scales of risk–benefit in the recipient’s favor, clinically oriented studies that unveil patient-specific risk factors have become crucial.

In light of these unmet needs, this study aimed to: (1) report the crude incidence rate of GBS following SARS-CoV-2 vaccination, (2) evaluate how vaccine dose, mechanism, age, and sex may affect the risk of GBS following SARS-CoV-2 vaccination, and (3) clinically compare head-to-head GBS cases following two major mechanisms of vaccines. We believe that our study can validate previous large-scale database-driven studies and provide a neurologist’s perspective on real-world data.

## Methods

### Study design and population data of vaccinated inhabitants

We conducted a prospective regional surveillance study for the occurrence of GBS in the Gyeonggi Province, South Korea, from February 26th, 2021, to March 15th, 2022.

The Gyeonggi Province is one of the largest local government bodies in South Korea, inhabited by approximately 13 million people, almost one-third of the nation’s population. As of March 2022, 85.9% of its residents had been fully vaccinated and 86.8% had been vaccinated at least once against SARS-CoV-2. Owing to its large population, the Gyeonggi Province has served as a centerpiece for the surveillance of adverse events following SARS-CoV-2 vaccination in South Korea.

The Gyeonggi province population data on vaccine dose (first, second, and third), mechanism (mRNA-based versus viral vector-based), age, and sex for individual vaccination events (per dose) were mined and reconstructed from a centralized, conjoined database formed by 48 community health centers in charge of SARS-CoV-2 vaccinations performed in designated vaccination centers, hospitals, and nursing homes in the respective city jurisdictions. The final merged dataset was used for statistical analysis.

### Passive AEFI/AESI surveillance and data collection of GBS cases

From the start of the nationwide vaccine rollout, as part of a government led passive surveillance program, patients and physicians were asked to report relevant AEFI/AESI to the local government authorities for post-marketing survey of SARS-CoV-2 vaccines. The Korean government motivated both physicians and patients with expert feedback and monetary compensation in case of a plausible association between the reported adverse event and the vaccine. Additionally, in an effort to overcome the shortcomings of passive surveillance, Gyeonggi Province has periodically monitored reporting rates in all province hospitals and issued updated education resources to in-hospital infection control centers to aid in prompt reporting of cases.

**Figure 2:**
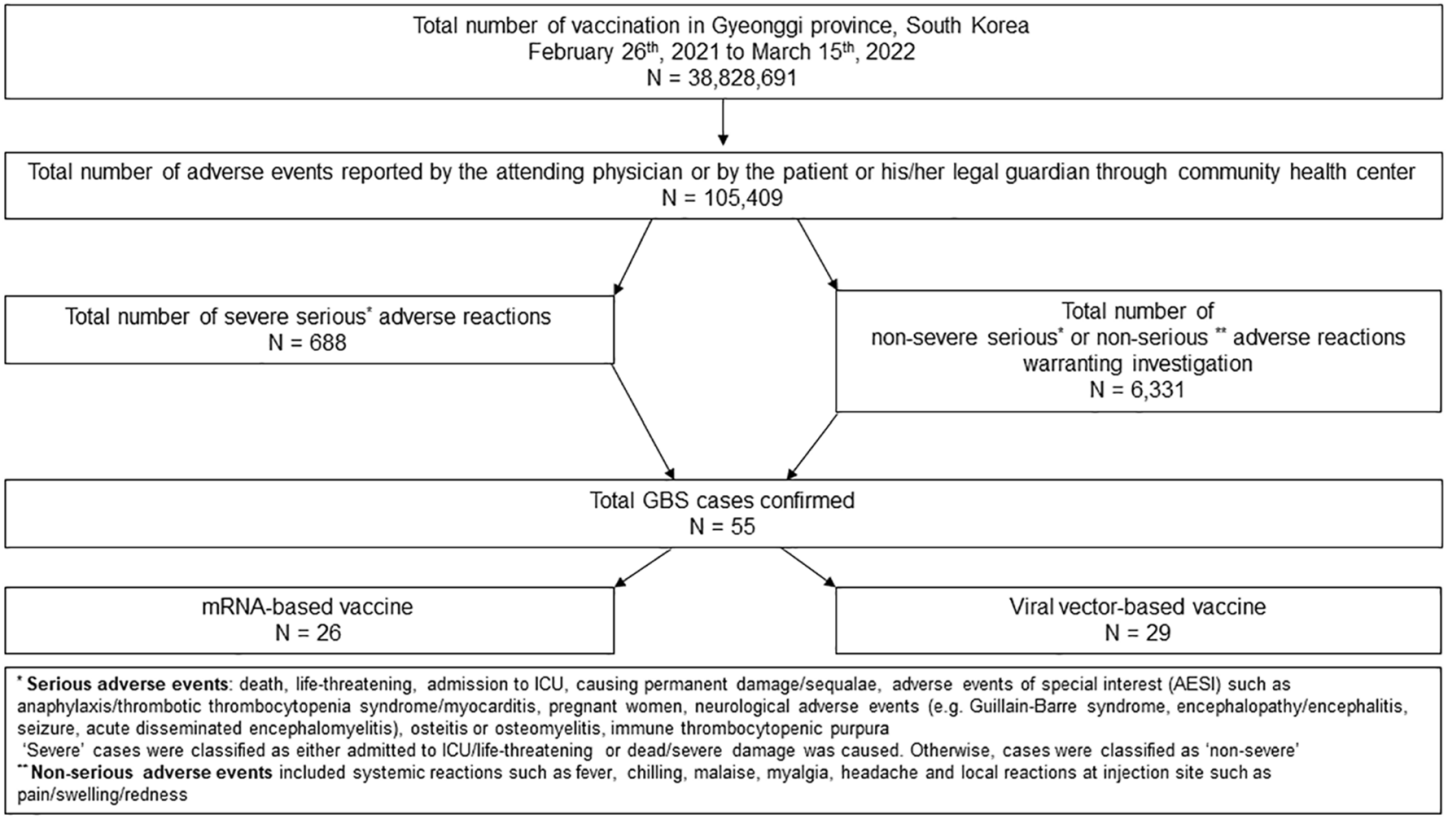
Flow diagram of study participants.

The Gyeonggi Infectious Disease Control Center has dealt with all reported individual cases of AEFI/AESI following SARS-CoV-2 vaccination, including GBS, and reviewed electronic medical records from hospitals and drug utilization review (DUR) records provided by the Korean Health Insurance Review and Assessment Service. It also interviewed the patient or the primary caregiver, and engaged in discussions with the relevant medical personnel to arrive at a conclusion on the diagnosis. The adverse events reported by the patient, their legal guardian, or the attending physician were classified as serious or non-serious, and serious adverse events were classified as severe or non-severe (Fig. 2). The validity of the diagnoses was confirmed through weekly expert consensus meetings. Cumulative data from the individual reports were pooled and reviewed.

Upon gathering relevant cases, we included patients diagnosed with GBS within 42 days after the last dose of SARS-CoV-2 vaccine. Also, we excluded the following participants from the study: (1) aged less than 12 years, (2) cases in which other vaccinations (e.g., seasonal influenza, hepatitis B) were administered within 3 months, (3) cases with relevant competing causes of paraparesis or quadriparesis, (4) cases with previous SARS-CoV-2 infection, and (5) previous diagnosis of GBS. In total, two patients satisfying the third criterion due to compressive myeloradiculopathy and myelopathy were excluded from the study.

From a total of 38,828,691 SARS-CoV-2 vaccine doses administered from February 26th, 2021, to March 15th, 2022, 105,409 adverse events were reported. A total of 688 cases had severe adverse reactions and 6331 cases had non-severe serious or non-serious adverse events, warranting further investigation. A total of 55 cases of GBS post SARS-CoV-2 vaccination were identified, as illustrated in Fig. 2. The electrophysiological data were available for 33 cases.

### Review of pooled data and GBS diagnosis

The Brighton Collaboration Case Definition^21^ and NINDS GBS criteria^22^ for classic sensorimotor types, as well as the panel suggested by Leonhard et al.^23^ for GBS variant subtypes, were used to reconfirm the validity of the diagnosis of GBS with temporal association to SARS-CoV-2 vaccination. The raw data of nerve conduction studies (NCS) were reviewed using the criteria proposed by Rajabally et al.^24^ Two neurology experts were involved in confirming the validity of the GBS diagnosis and the classification of clinical and electrodiagnostic subtypes.

Moreover, for a deeper understanding of the clinical course and characteristics of GBS following SARS-CoV-2 vaccination, we collected data on the time from vaccination to symptom onset, laboratory findings such as CSF leukocyte count and protein level, anti-ganglioside antibodies (anti-GM1 IgG, anti-GM1b IgG, and anti-GQ1b IgG), markers of severity, such as GBS disability scale (Hughes scale) at nadir and at 1 month post-treatment, Medical Research Council (MRC) sum score at nadir, ICU stay, mechanical ventilator requirement, and death.

### Statistical analyses

In this study, descriptive statistics were determined according to data attributes. Continuous data are displayed as means with standard deviations or medians with ranges, whereas categorical variables are displayed as absolute and relative frequencies. We analyzed the differences between the groups (mRNA-based versus viral vector-based) using Student’s *t* test or Mann–Whitney U test for continuous variables and Chi-square test or Fisher's exact test for categorical variables.

The incidence rate (IR) used in our study was defined as the number of new GBS adverse events per million doses of vaccines administered during the study period^13,25,26^. The 95% confidence intervals (CIs) for the IR were estimated using the Fay-Feuer method for gamma-based central CIs for directly standardized rates^27^. Poisson regression analysis was performed considering dose (first, second, or third), vaccine mechanism (mRNA-based or viral vector-based), age (< 30, 30–59, or ≥ 60), and sex (women or men) variables, and it was not significant (*p* = 0.9589) in the over-dispersion test. The results were expressed as the incidence rate ratio (IRR) and 95% CI. All statistical analyses were performed using a two-sided test and were considered statistically significant at a significance level of 0.05. Statistical analyses were performed using IBM SPSS Statistics (version 24.0; IBM, Armonk, NY, USA) and Rex (version 3.6.0, RexSoft Inc., Seoul, Korea). Forest plots were drawn using the R statistical software program (version 4.1.2; R Core Team 2021).

### Declarations

#### Ethics approval and consent to participate

The Korean Public Institutional Review Board granted exemption for this study because it involved analysis of de-identified data already obtained through epidemiological investigation, presented minimal risk to the participants, and met the needs of the current public health interest (identifier: P01-202204-01-006). Informed consent was obtained from all subjects and/or their legal guardian(s). All methods were carried out in accordance with relevant guidelines and regulations.

## Results

### Incidence rate of GBS post SARS-CoV-2 vaccination

**Figure 3:**
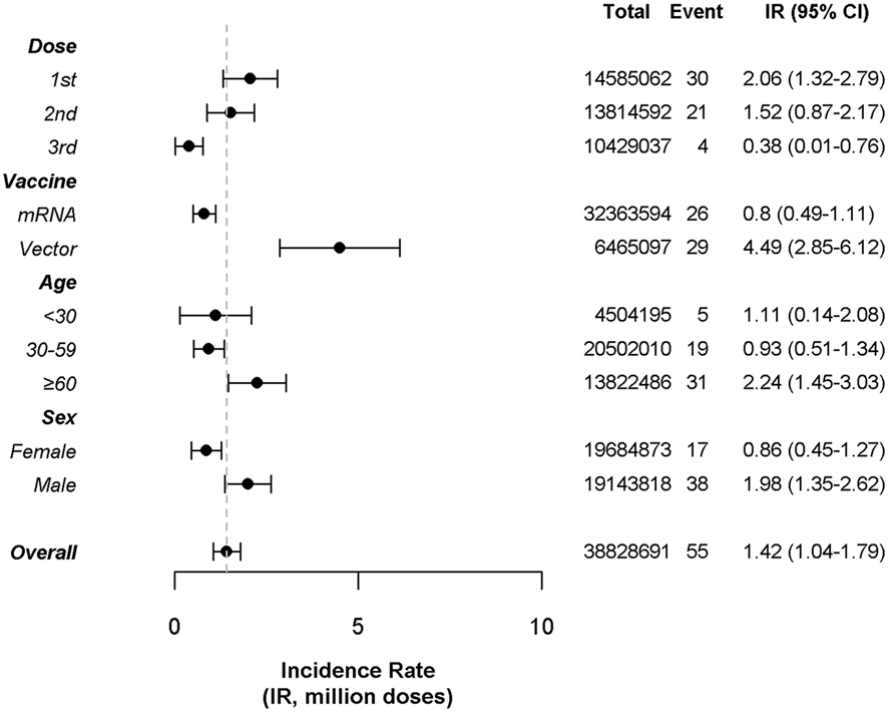
Incidence rate of GBS following SARS-CoV-2 vaccination, stratified by number of doses, vaccine types, age group and sex. Total doses of each vaccines administered, the number of events reported, and the incidence rate (IR) of GBS per dose, vaccine mechanism, age group, and sex are illustrated here.

A total of 38,828,691 doses were administered in the Gyeonggi province, and the overall incidence of GBS following SARS-CoV-2 vaccination was 1.42 per million doses (95% CI 1.04–1.79). The incidence of GBS following SARS-CoV-2 vaccination decreased from the first (IR, 2.06; 95% CI 1.32–2.79) to third dose (IR, 0.38, 95% CI 0.01–0.76). Upon evaluation based on the mechanism of vaccine, the incidence rate of GBS after viral vector-based vaccines was 4.49 per million doses (95% CI 2.85–6.12), higher than that after mRNA-based vaccines, which was 0.80 per million doses (95% CI 0.49–1.11). In terms of age, people aged 60 years or above exhibited a higher incidence of GBS (IR 2.24; 95% CI 1.45–3.03), compared to younger age groups (IR, 1.11; 95% CI 0.14–2.08). Men showed a higher incidence of GBS (IR, 1.98, 95% CI 1.35–2.62) compared to women (IR, 0.86; 95% CI 0.45–1.27) (Fig. 3).

### Risk factors for GBS following SARS-CoV-2 vaccination

In univariate analysis, both the second and third doses of the vaccines tended to reduce the risk of GBS from the first, but only the third dose demonstrated a statistically significant decrease in risk (crude IRR, 0.187; 95% CI 0.066–0.529). People who received viral vector-based vaccines were significantly more likely to develop GBS than those who received mRNA-based vaccines (crude IRR, 5.584; 95% CI 3.289–9.480). There was no statistically significant difference by age group (crude IRR 0.835; 95% CI 0.312–2.236 for the 30–59 age group, crude IRR 2.020; 95% CI 0.786–5.196 for the ≥ 60 age group). Men were more likely to develop GBS following SARS-CoV-2 vaccination than were women (crude IRR, 2.299; 95% CI 1.297–4.072). Finally, as a result of multivariate analysis, only dose (adjusted IRR 0.804; 95% CI 0.458–1.411 for second, adjusted IRR 0.331; 95% CI 0.110–0.997 for third), vaccine mechanism (adjusted IRR 3.745; 95% CI 1.979–7.087), and sex (adjusted IRR 2.285; 95% CI 1.289–4.051) showed statistically significant differences in the incidence of GBS (Table 1).

**Table 1:** Incidence rate ratios for SARS-CoV-2 vaccination and GBS following vaccination.

	Univariable analysis				Multivariable analysis			
	IRR	Lower	Upper	*P* value	IRR	Lower	Upper	*P* value
Dose								
1st	1				1			
2nd	0.739	0.423	1.291	0.288	0.804	0.458	1.411	0.447
3rd	0.187	0.066	0.529	0.002	0.331	0.110	0.997	0.049
Vaccine mechanism								
mRNA	1				1			
Viral vector	5.584	3.289	9.480	< 0.001	3.745	1.979	7.087	< 0.001
Age (years)								
< 30	1				1			
30–59	0.835	0.312	2.236	0.720	0.718	0.265	1.946	0.515
≥ 60	2.020	0.786	5.196	0.145	1.083	0.387	3.032	0.880
Sex								
Women	1				1			
Men	2.299	1.297	4.072	0.004	2.285	1.289	4.051	0.005

*IRR* incidence rate ratio.

### Clinical characteristics of GBS following SARS-CoV-2 vaccination

The mean age of GBS patients was 57.6 years (SD: 17.3); 38 (69.1%) were men, and 17 (30.9%) were women. The mean time from vaccination to symptom onset was 18.2 days (SD: 16.2) days. Rarer clinical phenotypes were observed, but classic sensorimotor and pure motor phenotypes prevailed (N = 23, 41.8% and N = 23, 41.8%, respectively). Electrodiagnostic classification revealed 20 demyelinating types (60.6%) and 7 axonal types (21.2%). Two patients in the viral vector-based vaccine group were positive for either GM1 or GD1b IgG. Albuminocytologic dissociation was observed in all cases in which CSF analysis was available. The median MRC sum score at nadir was 46.0 (IQR: 33.0–54.0), and the GBS disability scale at nadir was 4.0 (IQR: 2.8–4.0). Of the nine (16.7%) patients who needed ICU care, seven (13%) required mechanical ventilation and among them, five (9.1%) died (Table 2).

**Table 2:** Clinical characteristics and relevant measurements in post-vaccination GBS cases.

	Total (N = 55)	mRNA-based (N = 26)	Viral vector-based (N = 29)	*P* value
Age (years)	57.6 ± 17.3	56.6 ± 22.0	58.4 ± 11.9	0.718
Sex, N (%)				0.545
Men	38 (69.1)	19 (73.1)	19 (65.5)	
Women	17 (30.9)	7 (26.9)	10 (34.5)	
Dose number, N (%)				0.022
1st	30 (54.5)	10 (38.5)	20 (69.0)	
2nd	21 (38.2)	12 (46.2)	9 (31.0)	
3rd	4 (7.3)	4 (15.4)	0 (0.0)	
Time from vaccination-to-symptom onset (d)	18.2 ± 16.2	14.7 ± 14.6	21.3 ± 17.2	0.135
Clinical phenotype, N (%)				0.525
Classic sensorimotor	23 (41.8)	10 (38.5)	13 (44.8)	
Pure motor	23 (41.8)	12 (46.2)	11 (37.9)	
Paraparetic	2 (3.6)	0 (0.0)	2 (6.9)	
Pharyngeal-cervical-brachial	1 (1.8)	1 (3.8)	0 (0.0)	
BFP	1 (1.8)	0 (0.0)	1 (3.4)	
Miller–Fisher syndrome	2 (3.6)	1 (3.8)	1 (3.4)	
Overlap syndrome	1 (1.8)	0 (0.0)	1 (3.4)	
Undetermined	2 (3.6)	2 (7.7)	0 (0.0)	
Anti-ganglioside antibody, N (%)				0.486
Anti-GM1 IgG or GD1b IgG	2 (5.4)	0 (0.0)	2 (5.4)	
Anti-GQ1b IgG	0 (0.0)	0 (0.0)	0 (0.0)	
CSF profile				
WBC (/μl)	2.0 ± 4.2	2.0 ± 5.0	2.1 ± 3.0	0.938
Protein (mg/dl)	61.9 ± 35.4	61.5 ± 34.7	62.4 ± 37.4	0.933
EDX classification, N (%)^a^				0.793
Demyelinating	20 (60.6)	9 (60.0)	11 (61.1)	
Axonal	7 (21.2)	4 (26.7)	3 (16.7)	
Equivocal	6 (18.2)	2 (13.3)	4 (22.2)	
Severity and prognosis				
Mechanical ventilator requirement, N (%)	7 (13.0)	5 (19.2)	2 (7.1)	0.243
ICU stay, N (%)	9 (16.7)	6 (23.1)	3 (10.7)	0.286
GBS disability scale at nadir	4.0 (2.8–4.0)	4.0 (3.0–4.0)	4.0 (2.0–4.0)	0.420
MRC sum score at nadir	46.0 (33.0–54.0)	47.0 (32.3–54.0)	45.5 (34.5–54.3)	0.992
GBS disability scale at 1-month post-treatment	3.0 (2.0–4.0)	3.0 (2.0–4.0)	2.0 (1.0–4.0)	0.285
Δ GBS disability scale (nadir—1 month post-treatment)	1.0 (0.0–1.0)	1.0 (0.0–1.0)	1.0 (0.0–1.0)	0.695
Death, N (%)	5 (9.1)	2 (7.7)	3 (10.3)	1.000

Continuous data are presented as the mean ± SD; clinical scales are presented as medians (interquartile range).

*BFP* bifacial weakness with distal paresthesia, *CSF* cerebrospinal fluid, *EDX* electrodiagnostic, *ICU* intensive care unit, *MRC* Medical Research Council.

^a^Electrodiagnostic classification was performed for 33 patients whose raw nerve conduction study data were available.

There were no significant differences between the groups based on the mechanism of vaccines (mRNA-based and viral vector-based). Classic sensorimotor and pure motor subtypes were the predominant clinical phenotypes for both mRNA-based and viral vector-based vaccines, and there were no statistically significant differences in these phenotypes (*p* = 0.525). Demyelinating type was the dominant electrodiagnostic subtype in both the mRNA-based and viral vector-based vaccines, and there were no statistically significant differences in electrodiagnostic classification (*p* = 0.793). Nevertheless, GBS following mRNA-based vaccines was associated with later doses, whereas GBS following viral-vector-based vaccine was associated with the initial dose (*p* = 0.022). In addition, a higher number of mechanical ventilator requirements and ICU stay were observed for mRNA-based vaccines than for viral vector-based vaccines, but the difference was not statistically significant (*p* = 0.243 and *p* = 0.286, respectively). In terms of motor symptom severity at the nadir, no significant difference between the two vaccine mechanisms was detected for the GBS disability scale and MRC sum scale (*p* = 0.420 and *p* = 0.992, respectively). For prognostics, no significant difference between the two vaccine mechanisms were detected for GBS disability scale at 1-month post-treatment and number of deaths (*p* = 0.285 and *p* = 1.000).

## Discussion

We performed a large population-based prospective surveillance study featuring 38 million doses of the SARS-CoV-2 vaccine. Specifically, we meticulously reviewed each reported GBS case following SARS-CoV-2 vaccination; comprehensively assessed the clinical characteristics; and evaluated the contribution of variables such as vaccine dose, mechanism, age, and sex in developing GBS to improve internal validity and provide a clinical perspective. We believe our study is the first to concomitantly provide a broad view of the occurrence of GBS in a large, vaccinated population and delve deeper into each confirmed case. The key findings of this study are as follows: (1) GBS following SARS-CoV-2 vaccination was not clinically distinct from GBS pre-dating the COVID-19 pandemic; (2) viral vector-based vaccines were associated with a higher risk of developing GBS; and (3) the third dose of vaccine was associated with a lower risk of developing GBS.

In terms of the demographic and clinical characteristics of GBS post SARS-CoV-2 vaccination, they do not differ significantly from independent cases of GBS. First, men were associated with an increased risk of GBS following SARS-CoV-2 vaccination compared with women. This finding has previously been reproduced in large-scale epidemiological studies, regardless of vaccination^28,29^. Despite reports of diverse clinical subtypes, classic sensorimotor and pure motor types remain the most common. Likewise, when nerve conduction studies were reviewed, the demyelinating type was the most common electrodiagnostic subtype; these results are in accordance with previous epidemiological studies of the pre-pandemic era^30^. Notably, bifacial weakness with distal paresthesia subtypes increased in India and the UK following SARS-CoV-2 vaccination^31,32^, but a large-scale epidemiological study on the ChAdOx1nCoV-19 vaccine demonstrated no significant increase in these rare subtypes^14^. We also performed an in-depth analysis of clinical symptoms and signs of the GBS patients at initial visit and nadir, to address this concern (Supplementary Table S1). Among the five early facial palsy patients, four patients had isolated cranial nerve involvement; at nadir however, one patient developed pure motor type GBS and two patients developed classic sensorimotor GBS. Only one patient who received a viral vector-based vaccine was found to have BFP variant GBS at nadir. Additionally, in the same analysis, mRNA-based vaccine group showed lower extremity dominant involvement, whereas viral vector-based vaccine group showed equal involvement of upper and lower limbs at nadir—the clinical significance of this finding however, remains unclear. In fact, we might be dealing with the same kind of demon; only the trigger had changed. GBS following SARS-CoV-2 vaccination is not a clinically distinct type because there were no significant differences in age, sex, latency to symptom onset, clinical phenotype, electrodiagnostic subtype, severity, or prognosis when stratified by vaccine mechanisms (mRNA-based versus viral vector-based).

The incidence rate of post-vaccination GBS in our study was 4.49 per million doses of viral vector-based vaccines and 0.8 per million doses of mRNA-based vaccines. Our results follow the same propensity as those from a nationwide, database-driven study on GBS following SARS-CoV-2 vaccination, which has reported a markedly high incidence rate of GBS following the Ad26. COV2.S vaccine but a much lower incidence rate for mRNA-based vaccines^13^.

Upon assessing the risk factors for GBS post SARS-CoV-2 vaccination, the viral vector-based vaccine was associated with a three-to-four fold increased risk of GBS compared to mRNA-based vaccines, a finding consistent with previous studies^13,14,20^. GBS following the viral vector-based vaccine was associated with the initial dose, while GBS following the mRNA vaccine was associated with the latter doses (second and third). This finding parallels the reactogenicity of each vaccine mechanism; BNT162b2 and mRNA-1273 vaccines exhibited increased reactogenicity after the second and third doses of vaccination^33–36^, while the ChAdOx1nCoV-19 vaccine exhibited increased reactogenicity after the first dose of vaccination^37^. As an analogy, a similar result has been observed for vaccine-associated myocarditis, where an increased incidence of vaccine-associated myocarditis has been reported in the younger male population after the second dose of mRNA-based vaccination^38^. Hence, this finding may support the hypothesis that GBS following vaccination has a plausible immunological basis. A possible—but not yet firmly elucidated—mechanism behind the immunopathogenesis of GBS may be a complex interplay between CD4^+^/CD8^+^ T-cells and B-cells; it is induced primarily by innate immunity and T-cell response and followed by a progressive nerve injury due to humoral immunity^39,40^. At a molecular level, SARS-CoV-2 vaccines induce Th1 skewed immune response post vaccination^41,42^, inducing the release of cytokines such as IFN-γ and IL-2. IFN-γ is elevated in the acute phase of GBS^43^ and responsible for recruiting primed macrophages. IL-2 is responsible for sustaining both cell- and antibody-mediated humoral immunity inducing clonal expansion of both B- and T-cells^39^. While there is no tangible evidence of molecular mimicry between vaccine-targeted proteins (e.g. spike protein) and myelin, paranode, or nodal proteins yet, the possibility should be addressed in future studies.

Finally, the third dose, or a booster dose of the SARS-CoV-2 vaccine, was associated with a lower risk of GBS. This finding can be attributed to several factors; first and foremost, a combination of a decrease in booster shot recipients and attrition of an already susceptible population following the first and second doses. Second, mRNA-1273 booster shots were carried out using half the usual dose (0.5 mL/100 µg to 0.25 mL/50 µg). As a smaller amount of substrate for the immune response was administered into the recipient’s blood, it is possible that the resulting immune reaction was weaker than that after the previous doses, potentially reflecting a dose–response relationship. However, in our population, mRNA-1273 vaccine doses accounted for only up to 3% of the whole vaccination; therefore, the effect of this modification in the protocol may not suffice to explain our findings and a national-scale analysis involving a larger population is required to validate this hypothesis. Lastly, there has been an absolute decrease in the number of booster shot recipients since October 2021, largely due to the increase in public COVID-19 risk tolerance and vaccine hesitancy. Furthermore, these booster shots were mostly mRNA-based vaccines, which, as previously discussed, were associated with a lower risk of GBS following vaccination. Nevertheless, this finding directly opposes previous results on immunogenicity and reactogenicity of the third dose of vaccines, as studies have revealed augmented immunogenicity after the booster shot (higher IgG levels compared to the second dose) in both types of vaccines^37,44^, and reactogenicity in mRNA-based vaccines^35,36,45^. Therefore, this finding should be interpreted with caution.

This study had several limitations that need to be acknowledged. First, although the temporal association suggested in our study may provide valuable insight into causality assessment, an observational study inherently discovers only associations and not causality. Additionally, since our population data were collected per vaccine dose, it is difficult to directly compare our results with previous epidemiological studies which mostly report incidence by per person basis. Nevertheless, the total incidence of GBS after SARS-CoV-2 vaccination in our study is comparable to the numbers reported by García–Grimshaw and colleagues, where they observed an incidence of 1.19/1,000,000 doses^26^. To conclude the risk of GBS from SARS-CoV-2 vaccination, further studies with incidence per person might compare directly with the incidence before vaccination. Regardless, the selection bias was minimal because our study was based on a large community population with more than 38 million doses. Although our study was not performed in a carefully controlled setting and potential unidentified confounders may still exist, we show real-world data of a heterogeneous, unselected population, a strength rather than a weakness in assessing the incidence rate and associated risks. Second, the prospective surveillance design is vulnerable to under-reporting bias. However, in order to increase sensitivity, we undertook efforts to overcome this limitation as described in the method section. Third, concurrent infections could have been better addressed using viral and bacterial panel in these patients. However, these patients did not present with symptoms suggestive of infection and SARS-CoV-2 infection was ruled-out in all GBS patients upon admission to the hospital regardless. Finally, we did not account for the differences between homologous and heterologous vaccination and the different immunogenicity and reactogenicity conveyed by each method^46,47^. In our study, only one case of GBS following SARS-CoV-2 vaccination presented after heterologous vaccination (received ChAdOx1-S/nCoV-19 for the first dose and BNT162b2 for the second dose). Future studies should focus on whether increased immunogenicity and reactogenicity after heterologous vaccination increase the risk of GBS and other immune-mediated adverse events.

In conclusion, GBS following SARS-CoV-2 vaccination is a growing concern as some cases may lead to significant morbidity or even mortality. It is imperative that physicians closely monitor patients following SARS-CoV-2 vaccination, especially men who are vaccinated with an initial dose of viral-vector-based vaccine. Future studies should focus on integrating clinical and large epidemiological perspectives to generate an individualized risk in taking SARS-CoV-2 vaccination to recommend the most suitable type of vaccine with the lowest risk of adverse events. Furthermore, countries should try to foster pharmacovigilance on immune-mediated subacute-onset neurological complications and try to provide clearer, digestible information to the public to guide upcoming vaccination campaigns.

## Supplementary Information

Supplementary Information.

## Data Availability

The datasets generated and/or analysed during the current study are not publicly available due to the sensitive nature of the data and patient confidentiality, but the derived data are available from the corresponding author on reasonable request.
