# Supplementary material for: Real-world data on the incidence and risk of Guillain–Barré syndrome following SARS-CoV-2 vaccination: a prospective surveillance study
Source: Sci Rep. 2023 Mar 7;13:3773. doi: 10.1038/s41598-023-30940-1 (PMC9989583; doi:10.1038/s41598-023-30940-1)
Supplement: Supplementary file 1 — Supplementary Information. [file 41598_2023_30940_MOESM1_ESM.pdf]

**Supplementary Table S1. Temporal evolution of clinical signs and symptoms of GBS following SARS-CoV-2 vaccination from initial presentation to nadir**

| | Initial presentation | | | P-value[Ŧ] | Nadir | | | P-value[Ŧ] |
|---|---|---|---|---|---|---|---|---|
| | Total | mRNA-based | Viral vector-based | | Total | mRNA-based | Viral vector-based | |
| Type of symptom | | | | 0.341 | | | | 0.344 |
| Motor only | 25 (45.5) | 12 (46.2) | 13 (44.8) | | 13 (24.1) | 8 (32.0) | 5 (17.2) | |
| Sensory only | 15 (27.3) | 5 (19.2) | 10 (34.5) | | 0 (0.0) | 0 (0.0) | 0 (0.0) | |
| Sensorimotor | 15 (27.3) | 9 (34.6) | 6 (20.7) | | 41 (75.9) | 17 (68.0) | 24 (82.8) | |
| Dominance pattern (upper vs. lower)[*] | | | | 0.860[Ꝁ] | | | | -0.012[Ꝁ] |
| Upper extremity dominant | 16 (32.0) | 8 (33.2) | 8 (30.8) | | 3 (3.8) | 2 (8.7) | 0 (0.0) | |
| Lower extremity dominant | 26 (52.0) | 13 (54.2) | 13 (50.0) | | 21 (40.4) | 13 (56.5) | 8 (27.6) | |
| Equally dominant | 8 (16.0) | 3 (12.5) | 5 (19.2) | | 28 (53.8) | 8 (34.8) | 20 (69.0) | |
| Dominance pattern (proximal vs. distal)[*] | | | | 1.000[Ꝁ] | | | | 0.842[Ꝁ] |
| Proximal dominant | 6 (13.0) | 3 (14.3) | 3 (12.0) | | 11 (22.0) | 4 (18.2) | 7 (25.0) | |
| Distal dominant | 32 (69.6) | 14 (66.7) | 18 (72.0) | | 20 (40.0) | 9 (40.9) | 11 (39.3) | |
| Equally dominant | 8 (17.4) | 4 (19.0) | 4 (16.0) | | 19 (38.0) | 9 (40.9) | 10 (35.7) | |
| Cranial nerve involvement[§] | 10 (18.9) | 5 (20.0) | 5 (17.9) | 1.000 | 22 (42.3) | 13 (54.2) | 9 (32.1) | 0.160 |
| Ophthalmoplegia | 2 (3.7) | 2 (7.7) | 0 (0.0) | 0.227 | 2 (3.8) | 2 (8.3) | 0 (0.0) | 0.200 |
| Hypoglossal nerve palsy | 1 (1.9) | 1 (3.8) | 0 (0.0) | 0.481 | 1 (1.9) | 1 (4.2) | 0 (0.0) | 0.453 |
| Trigeminal nerve palsy[‡] | 0 (0.0) | 0 (0.0) | 0 (0.0) | - | 1 (1.9) | 0 (0.0) | 1 (3.4) | 1.000 |
| Bulbar palsy | 8 (14.8) | 4 (15.4) | 4 (14.3) | 1.000 | 15 (28.3) | 9 (37.5) | 6 (20.7) | 0.227 |
| Facial nerve palsy[†] | 5 (9.3) | 2 (7.7) | 3 (10.7) | 1.000 | 10 (18.9) | 6 (25.0) | 4 (13.8) | 0.482 |
| Symmetry[*] | | | | 0.381 | | | | 0.441 |
| Symmetrical | 38 (69.1) | 22 (75.9) | 16 (61.5) | | 44 (84.6) | 18 (78.3) | 26 (89.7) | |
| Asymmetrical | 17 (30.9) | 7 (24.1) | 10 (38.5) | | 8 (15.4) | 5 (21.7) | 3 (10.3) | |
| Modality of sensory symptoms[§] | | | | | | | | |
| Paresthesia | 24 (72.7) | 12 (75.0) | 12 (70.6) | 1.000 | 31 (73.8) | 11 (61.1) | 20 (83.3) | 0.159 |
| Hypesthesia | 9 (27.3) | 6 (35.3) | 3 (18.8) | 0.438 | 26 (63.4) | 12 (66.7) | 14 (60.9) | 0.754 |

| | | | | | | | | |
|---|---|---|---|---|---|---|---|---|
| Vibration | 1 (3.0) | 1 (6.3) | 0 (0.0) | 0.485 | 13 (31.7) | 6 (33.3) | 7 (30.4) | 1.000 |
| Proprioception | 3 (9.1) | 1 (6.3) | 2 (11.8) | 1.000 | 14 (34.1) | 6 (33.3) | 8 (34.8) | 1.000 |
| Pain, temperature | - | - | - | - | 9 (22.0) | 5 (27.8) | 4 (17.4) | 0.471 |
| Light touch | - | - | - | - | 11 (26.8) | 4 (22.2) | 7 (30.4) | 0.726 |
| Unspecified | 5 (15.2) | 1 (6.3) | 3 (23.5) | 0.335 | 5 (12.2) | 2 (11.1) | 3 (13.0) | 1.000 |
| Pain | 2 (6.1) | 2 (12.5) | 0 (0.0) | 0.227 | 7 (17.1) | 4 (22.2) | 3 (13.0) | 0.679 |

*Dominance (upper vs. lower, proximal vs. distal) and asymmetry was defined as 1 or more numerical difference in MRC grade

†At initial visit, only 1 patient had bilateral facial palsy, whereas at nadir, 8 patients developed bilateral facial palsies

‡1 patient complained of somatic numbness of the tongue without dysgeusia or motor dysfunction

§These findings are not mutually exclusive, since patients have complained of single or multisensory deficits

Ŧp-value by chi-squared test; ₭p-value by fisher's exact test
